# Immunological properties and protective efficacy of a single mycobacterial antigen displayed on polyhydroxybutyrate beads

**DOI:** 10.1111/1751-7915.12754

**Published:** 2017-07-17

**Authors:** Patricia Rubio‐Reyes, Natalie A. Parlane, Bryce M. Buddle, D. Neil Wedlock, Bernd H. A. Rehm

**Affiliations:** ^1^ Institute of Fundamental Sciences Massey University 11222 Private Bag Palmerston North New Zealand; ^2^ AgResearch, Hopkirk Research Institute Grasslands Research Centre 11008 Private Bag Palmerston North New Zealand; ^3^ Griffith Institute for Drug Discovery Griffith University Nathan QLD Australia

## Abstract

In 2015, there were an estimated 10.4 million new tuberculosis (TB) cases and 1.4 million deaths worldwide. Bacille Calmette–Guérin (BCG), an attenuated strain of *Mycobacterium bovis*, is the vaccine available against TB, but it is insufficient for global TB control. This study evaluated the immunogenicity of the *Mycobacterium tuberculosis* antigen Rv1626 in mice while assessing the effect of co‐delivering either Cpe30 (immunostimulatory peptide), CS.T3_378–395_ (promiscuous T helper epitope) or flagellin (TLR5 agonist) or a combination of all three immunostimulatory agents. Rv1626 and the respective immunostimulatory proteins/peptides were co‐displayed on polyhydroxybutyrate beads assembled inside an engineered endotoxin‐free mutant of *Escherichia coli*. Mice vaccinated with these beads produced immune responses biased towards Th1‐/Th17‐type responses, but inclusion of Cpe30, CS.T3_378–395_ and flagellin did not enhance immunogenicity of the Rv1626 protein. This was confirmed in a *M. bovis* challenge experiment in mice, where Rv1626 beads reduced bacterial cell counts in the lungs by 0.48 log_10_ compared with the adjuvant alone control group. Co‐delivery of immunostimulatory peptides did not further enhance protective immunity.

## Introduction

Polyhydroxyalkanoates (PHAs) are biopolyesters naturally produced as intracellular inclusions by many bacteria and archaea. The synthesis of the most common one, polyhydroxybutyrate (PHB), a short chain length PHA, involves three key enzymes, β‐ketothiolase (PhaA), acetoacetyl‐CoA reductase (PhaB) and polyester synthase (PhaC). The knowledge about topology, structure and properties of PhaC, which remains covalently linked to the PHB granules, has allowed translational fusion of foreign proteins to produce functionalized beads. These beads have been used for biomedical applications like protein production and purification as well as diagnostics and vaccines (Parlane *et al*., 2016; Rehm *et al*., 2016). Medical application was justified as beads are biocompatible and biodegradable, and their one‐step production of carrier and antigen is cost‐effective (Parlane *et al*., 2014).

An effective vaccine is needed for the global prevention of tuberculosis (TB). Such a vaccine needs to be affordable and accessible for developing countries, safe and able to elicit a longer lasting immunity than the current vaccine, Bacille Calmette–Guérin (BCG; Orme, 2013). PHB beads displaying Ag85A‐ESAT‐6 antigens from *Mycobacterium tuberculosis* induced stronger humoral and cellular immune responses than vaccination with soluble Ag85A‐ESAT‐6 (Grage *et al*., 2009) and resulted in protection against *Mycobacterium bovis* challenge (Parlane *et al*., 2012).

New‐generation adjuvants are being developed for subunit vaccines, for example molecular adjuvants, like promiscuous T helper epitopes that bind a number of HLA class molecules and allow entire populations to respond irrespective of MHC (Alexander *et al*., 2000). CS.T3_378–395_, a peptide from the circumsporozoite protein of *Plasmodium falciparum,* is a promiscuous T helper epitope almost universally recognized by human and mouse T lymphocytes (Kironde *et al*., 1991), producing immunological cross reactivity (Joshi *et al*., 2001) and potentiating immune responses (Kumar *et al*., 1999). One of the strongest pathogen‐associated molecular patterns (PAMPs) is flagellin, the monomeric subunit of the bacterial motility apparatus. Flagellin is the natural ligand of Toll‐like receptor (TLR) 5 and the only protein TLR agonist identified to date (de Zoete *et al*., 2010). Numerous studies have described the adjuvant properties of flagellin in the context of a broad range of recombinant vaccines (Mizel and Bates, 2010) inducing cellular responses, and humoral responses (Huleatt *et al*., 2007) even in a T‐cell‐restricted model (Bennett *et al*., 2015). Other peptides act as molecular adjuvants, like Cpe30 peptide which is derived from the C‐terminus 30 amino acids of *Clostridium perfringens* enterotoxin. Cpe30 binds to the protein claudin‐4, which is an endocytosis receptor on M cells (Ye *et al*., 2014). It has been used in the context of mucosal vaccination along with other antigens, inducing strong IgG1, IgG2a and IFN‐γ responses (Kakutani *et al*., 2010).

Previously, we demonstrated that beads displaying the Rv1626 antigen from *M. tuberculosis* were immunogenic, mediating an antigen‐specific antibody response (Rubio Reyes *et al*., 2016). Rv1626 interacts with the actin‐related protein 4 (ARP4) of mammalian cells, the function of this interaction is not clear, but it is possible that Rv1626 modulates the host cell cytoskeleton enabling *M. tuberculosis* survival and protection (Ghosh *et al*., 2013). There is strong production of Rv1626 in the early stages of *M. tuberculosis* growth (Haydel and Clark‐Curtiss, 2004), and it is constitutively produced and secreted (Sassetti *et al*., 2003).

In this study, the immunological properties of beads displaying Rv1626 and the molecular adjuvants, Cpe30, CS.T3_378–395_ or flagellin either alone or in combination was studied. The ability of these functionalized beads to protect mice against experimental challenge with *M. bovis* was determined.

## Results and discussion

### Development of *Escherichia coli* strains assembling PHA beads displaying various antigen Rv1626 and immunomodulators

**Figure 1:**
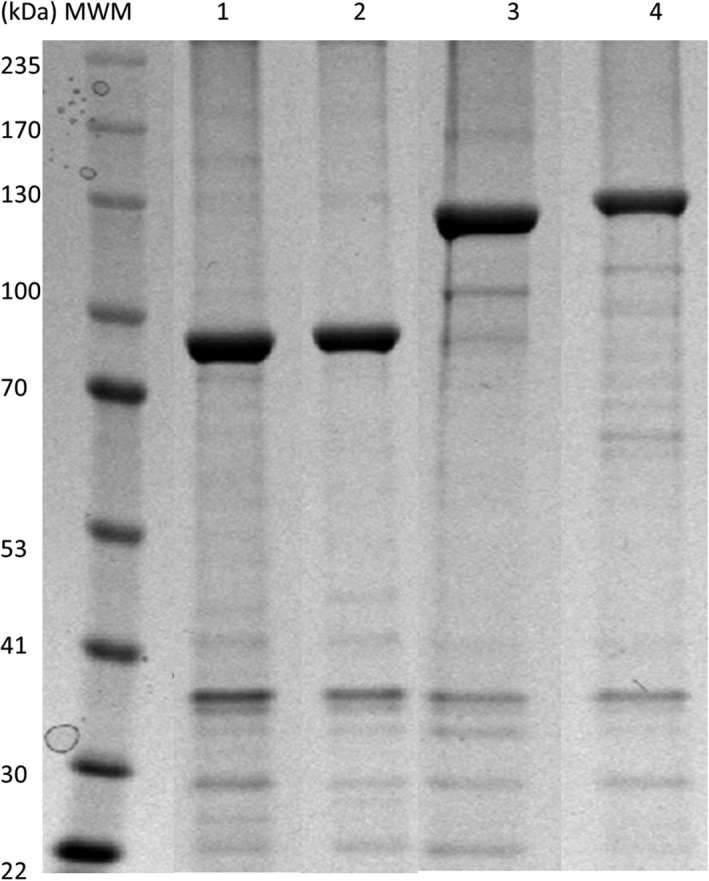
SDS–PAGE analysis of proteins attached to various polyester beads isolated from *Clear coli*. Lane 1, Cpe30‐Rv1626 beads (92.5 kDa); lane 2, CS.T3‐Rv1626 beads (93.1 kDa); lane 3, Fla66‐Rv1626 beads (134 kDa); lane 4, Cpe30‐CS.T3‐Fla66‐Rv1626 beads (141 kDa). Corresponding genes were inserted into pPOLYC‐Rv1626 plasmid using XhoI/BsrGI sites. The linker VLAVAIDKRGGGGG (hydrophobic‐charged amino acids) is included in this plasmid between PhaC and Rv1626 to facilitate display of the fusion partners (Jahns and Rehm, 2009). Proteins were quantified by SDS–PAGE gel densitometry.

Genes *cpe30, cs.t3*_*378–395,*_ flagellin_66*–494*_ and *cpe30‐cs.t3*_*378–395*_ *‐flagellin*_*66–494*_ were cloned separately upstream of *rv1626* into pPOLYC‐Rv1626 plasmid. *Clear coli* was transformed with the various plasmids and used as the host for producing PHA beads for vaccination of mice. Beads were purified, and bead protein profiles were analysed by SDS–PAGE (Fig. 1). A prominent band corresponding to the expected molecular weight of the full‐length fusion protein was observed in all cases and the identity of the band corresponding to PhaC‐Cpe30‐CS.T3‐Fla66‐Rv1626 was confirmed by MALDI‐TOF/MS. The amount of Rv1626 immobilized per mg of wet beads was 393 ng, 176.8 ng, 174 ng, 102 ng and 108 ng in Rv1626, Cpe30‐Rv1626, CS.T3‐Rv1626, Fla66‐Rv1626 and PhaC‐Cpe30‐CS.T3‐Fla66‐Rv1626 beads, respectively. There was 695 ng of PhaC in wild‐type beads. The various beads displaying the antigen and immunostimulatory molecules were used to study their immunogenicity in mice.

### Dose–response study

In order to choose a suitable dose of Rv1626 antigen to assess the ability of the various immunomodulators to enhance antigen‐specific immune responses, a dose–response study was conducted. Mice were vaccinated with different doses of Rv1626 (1.25, 2.5. 5 and 10 μg) displayed on beads and formulated in DDA (dimethyldioctadecylammonium). IgG1 and IgG2c responses in sera were analysed by ELISA (Fig. S1). IgG1 responses to Rv1626 antigen were significantly higher in mice vaccinated with 2.5 μg and 5 μg of Rv1626 beads than responses in mice vaccinated with wild‐type (Wt) beads or given DDA alone. No significant differences were observed between mice vaccinated with the different doses of Rv1626. IgG2c responses of mice vaccinated with Rv1626 beads did not increase with the dose, and responses were all significantly higher in mice vaccinated with 1.25 ‐ 10 μg Rv1626 than mice vaccinated with DDA alone. These results differ from the ones obtained by Parlane *et al*. (2009), where higher doses significantly increased the immune response (Grage *et al*., 2009). However, different antigens were used in these studies, and it is possible that even with the lower doses of Rv1626 the antibodies responses were already maximal. Based on these results, a dose of 2.5 μg Rv1626 on beads was chosen for further immunological studies.

### Determination of immunogenicity of Rv1626 beads

Serum IgG1 and IgG2c responses to Rv1626 in mice vaccinated with Rv1626 beads with or without addition of immunomodulators were evaluated by ELISA (Fig. S2). These responses were not significantly different between the groups but were increased compared to mice vaccinated with DDA alone. Mice splenocytes were stimulated with soluble Rv1626, and IL‐2, IL‐4, IL‐6, IL‐10, IL‐17A, IFN‐γ and TNF‐α levels were measured in culture supernatants (Fig. S3). Vaccination with the PHA beads triggered a Th1/Th17 skewed response, but inclusion of the molecular adjuvants did not increase the immunogenicity of Rv1626 compared to Rv1626 beads alone.

### Assessment of protective immunity induced by Rv1626 beads

As there are no confirmed correlates of protection for *M. tuberculosis* or *M. bovis* infection, the ability of Rv1626 displayed on beads and combined with immunomodulators Cpe30 or CS.T3 to protect against tuberculosis was evaluated in a *M. bovis* challenge experiment. These beads were selected from the immunogenicity experiment as they induced higher cytokines levels than the other vaccines.

**Figure 2:**
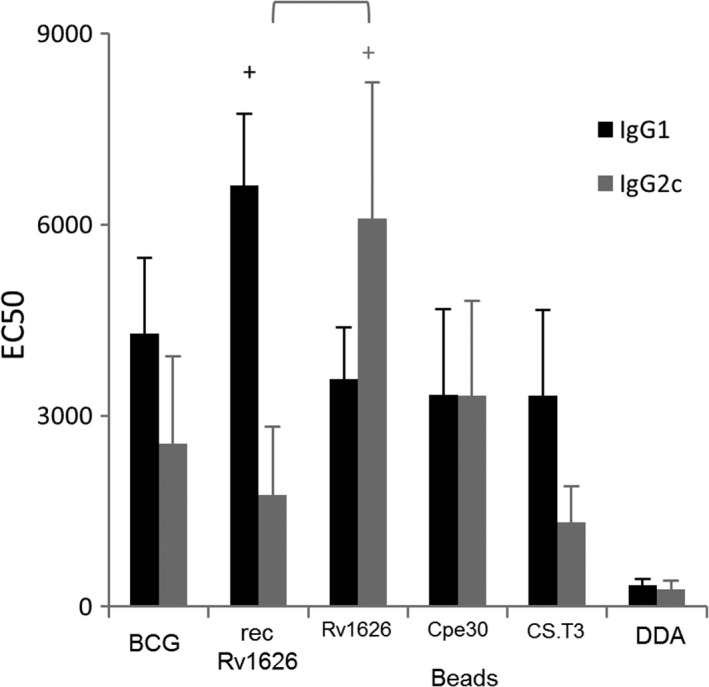
Serum IgG1 and IgG2c titres, expressed as EC50 values in mice (eight per group) subcutaneously vaccinated once with BCG, or three times at 9 day intervals with soluble recombinant Rv1626 (recRv1626), Rv1626 beads, Cpe30‐Rv1626 beads, CS.T3‐Rv1626 beads formulated with DDA or DDA alone. Three weeks after the final vaccination, the mice were euthanized and sera collected and analysed by ELISA using methods described previously by Rubio Reyes *et al*. (2016). Plates were incubated overnight at 4°C with 2.5 μg ml^−1^ of soluble recombinant Rv1626 obtained using a sortase‐based tagless protein purification system as previously described (Hay *et al*., 2015). EC50 was calculated adjusting to a 4‐parameters logistic curve the absorbance values (450 nm) corresponding to sera dilutions. EC50 is the mid‐point of the sigmoid (c coefficient of the curve). Values were analysed using a Dunn's test of multiple comparisons following a significant Kruskal–Wallis test. Each bar represents the mean values of 8 mice ± standard error of the mean, +, significantly greater than the DDA vaccinated group; groups between brackets, significantly different (*P* < 0.01).

Additional groups were given soluble recombinant Rv1626 or BCG while a control group received DDA. Antibody responses of mice vaccinated with the selected PHA bead‐based vaccines are shown in Fig. 2. Vaccination with soluble Rv1626 induced significantly higher levels of IgG1 than the negative control group while vaccination with Rv1626 beads higher levels of IgG2c than both the negative control group and the group vaccinated with soluble Rv1626.

The induced IgG subclasses provide an indication of the contribution of Th1‐ or Th2‐type cytokines in the response; thus, release of IgG1 is related to induction by Th2‐type cytokines, whereas production of IgG2c is related to induction by Th1‐type cytokines (Petrushina *et al*. 2003).

**Figure 3:**
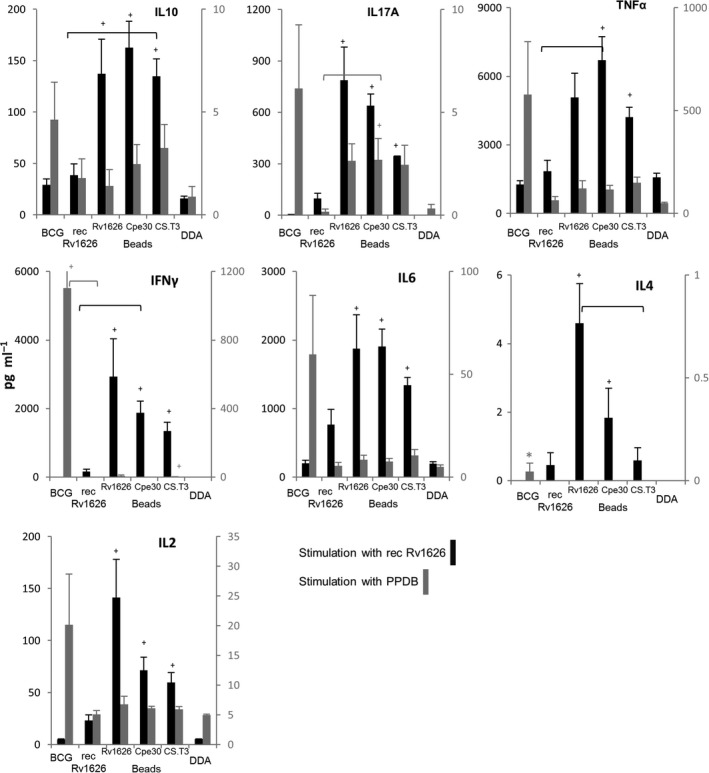
Cytokine responses of mice splenocytes upon stimulation with soluble recombinant Rv1626 (left axis) or PPDB (right axis; 5 μg ml^−1^). Mice (eight per group) were subcutaneously vaccinated once with BCG, or three times at 9 day intervals with either soluble recombinant Rv1626 (recRv1626), Rv1626 beads, Cpe30‐Rv2626 beads, CS.T3‐Rv1626 beads formulated with DDA or DDA alone. Three weeks after the last vaccination, mice were euthanized and splenocytes obtained and cultured as described by Parlane *et al*., 2012. Cytokine release was measured in the splenocyte culture supernatants using a BD CBA Mouse Th1/Th2/Th17 cytokine kit (BD Biosciences, USA). Values were analysed using a Dunn's test of multiple comparisons following a significant Kruskal–Wallis test. Each bar represents the mean of 8 mice ± standard error of the mean, +, significantly greater than DDA vaccinated group; *, significantly different from all groups, groups between brackets, significantly different (*P* < 0.01).

Splenocytes from mice were stimulated with 5 μg ml^−1^ of soluble recombinant Rv1626 or bovine PPD (purified protein derivative), and results are shown in Fig. 3. For splenocytes stimulated with soluble recombinant Rv1626, levels of IL‐10, IL‐17A, IL‐2, IL‐6 and IFN‐γ were significantly higher in mice vaccinated with Rv1626, Cpe30‐Rv1626 and CS.T3‐Rv1626 beads than in mice vaccinated with DDA alone. Mice vaccinated with Cpe30‐Rv1626 and CS.T3‐Rv1626 beads produced significantly higher TNF‐α levels than the negative control, and those vaccinated with Rv1626 and Rv1626‐Cpe30 beads produced higher levels of IL‐4. Differences were observed between mice vaccinated with beads and the soluble recombinant protein. The soluble Rv1626 induced significantly lower levels of TNF‐α and IFN‐γ than Cpe30‐Rv1626 beads and lower IL‐10 than CS.T3‐Rv1626 beads. For splenocytes stimulated with PPDB, BCG vaccination induced significantly higher levels of IFN‐γ than the negative control and higher IL‐4 than all the other groups.

Bacille Calmette–Guérin vaccination significantly reduced the bacterial burden in lungs compared with vaccination with soluble Rv1626, Cpe‐Rv1626, CS.T3‐Rv1626 beads or DDA and in spleen compared with all other groups (Table 1). Vaccination with BCG reduced bacterial cell counts in lungs and spleen by 1.35 log_10_ and 0.98 log_10,_ respectively, compared to the negative control (DDA). Vaccination with the Rv1626 beads reduced the lung bacterial counts by 0.48 log_10_. Although this reduction was not significant compared to the negative control, in this group, lung bacterial counts were not significantly different compared to those for the BCG‐vaccinated group. Derrick *et al*. (2013) reported a bacterial count reduction of 0.31 log_10_ after vaccination with soluble Rv1626 and challenge. In this study, soluble Rv1626 did not induce protection. Our data confirmed that the display of antigen on PHA beads increases induction of protective immunity compared to the soluble antigen as had been previously shown for other antigens (Parlane *et al*., 2012; Martinez‐Donato *et al*., 2016). It may be possible to achieve greater protection with Rv1626 beads, using the beads as a boost vaccine after primary vaccination with either BCG or ESAT6‐Ag85B. Derrick *et al*. (2013) achieved 1.12 log_10_ reduction after vaccinating mice with soluble Rv1626 following vaccination with ESAT6‐Ag85B.

**Table 1:** Vaccine induced protection in lung or spleen after *M. bovis* aerosol infection

Vaccine	Lung (log_10_) CFUs	Spleen (Log_10_) CFUs
BCG	4.83 ± 0.09 (−1.35)^b^	3.86 ± 0.05 (−0.98)^b^
rec Rv1626	6.22 ± 0.24 (+0.04)^a^	4.90 ± 0.12 (+0.06)^a^
Rv1626 beads	5.70 ± 0.04 (−0.48)^ab^	4.73 ± 0.08 (−0.11)^a^
Cpe‐Rv1626 beads	6.07 ± 0.09 (−0.12)^a^	4.72 ± 0.03 (−0.12)^a^
CS.T3‐Rv1626 beads	6.46 ± 0.38 (+0.28)^a^	5.06 ± 0.23 (+0.22)^a^
DDA	6.18 ± 0.37^a^	4.84 ± 0.19^a^

Mean count ± standard error of the mean (group mean‐DDA group mean). Female C57BL/6 mice (eight per group) were vaccinated once by the subcutaneous route with BCG Pasteur 1173P2 (10^6^ CFUs per animal) or three times at 9 day intervals with either soluble recombinant Rv1626 (rec Rv1626) or beads formulated with the adjuvant DDA (1.25 mg ml^−1^) or DDA alone (control group). Six weeks after the last vaccination, mice were aerosol challenged with *M. bovis* 83/6235 in a Madison chamber (calibrated to deliver 50 bacteria into the lungs) and five weeks later, euthanized and lungs and spleens homogenized and cultured. After three weeks of incubation, colonies were counted. Data were analysed using a Dunn's test of multiple comparisons following a significant Kruskal–Wallis test. Statistical significance is shown using a letter based system. Significantly different groups have different letters, and groups with means not significantly different share the same letter (*P* < 0.01).

**Figure 4:**
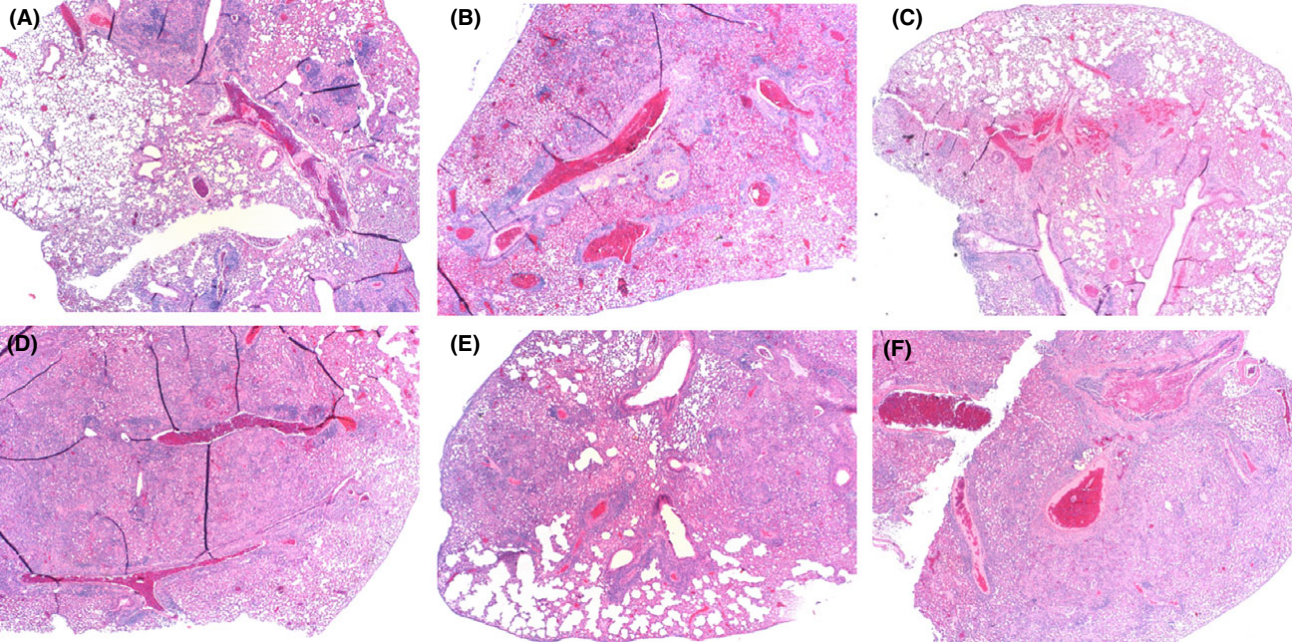
Histological appearance of lungs from mice that have the closest value to the CFUs mean after *M. bovis* challenge. Five weeks after challenge, mice were euthanized and lungs removed. Sections from the right apical lung were stained with H&E. Groups: (A), BCG; (B), soluble recombinant Rv1626; (C), Rv1626 beads; (D), Cpe30‐Rv1626 beads; (E), CS.T3‐Rv1626 beads; (F), DDA alone.

Lung sections were stained with haematoxylin and eosin (H&E; Fig. 4). Lungs from the adjuvant control mice had multiple, coalescing granulomas composed predominantly of epithelioid macrophages and lymphocytes. Intracellular, acid‐fast bacilli were observed in macrophages found in these lesions. The lungs of the BCG‐vaccinated mice had less lesion involvement. The lesions were composed of loosely organized accumulations of inflammatory cells within the perivascular areas, with a predominance of lymphocytes and fewer macrophages.

Assembling Cpe30 or CS.T3 with Rv1626 antigen on the same PHA bead did not significantly increase the immunogenicity of Rv1626 and may even have reduced protection. This could be due to a misfolding of the fusion protein on the beads when the immune modulators were included in the same fusion protein, interfering with exposure of Rv1626 conformational epitopes to cell receptors (Ahmad *et al*., 2016). The amount of immunomodulators on the various beads used in our study may not have been optimal as the primary concern was to have a similar dose of Rv1626 antigen in each type of vaccine. For example, too high levels of immunomodulator on the vaccine beads may have overstimulated cell receptors resulting in inflammation and tissue damage and impaired protective immunity (Xiao *et al*., 2015).

While further studies are needed to optimize the dose of immunomodulators on mycobacterial antigen displaying beads, this study demonstrated that a single *M. tuberculosis* antigen when displayed on PHA beads has the potential to protect against tuberculosis.

## Conflict of interest

B.H.A. Rehm is founding inventor, Chief Technology Officer and shareholder of PolyBatics Ltd that commercializes the PHA bead technology.

## Supporting information


**Fig. S1.** IgG1 and IgG2c titres, expressed as EC50 values in mice (8 per group) vaccinated subcutaneously three times at 9 day intervals with different doses of Rv1626 displayed on beads and Wt beads.
**Fig. S2.** Serum IgG1 and IgG2c titres, expressed as EC50 values in mice (8 per group) vaccinated subcutaneously three times at 9 day intervals with Rv1626 beads or Rv1626 beads displaying different immune modulators.
**Fig. S3.** Cytokine responses of mice splenocytes upon stimulation with soluble Rv1626 (recRv1626) and analysed by cytometry bead array.
