# Supplementary material for: Immunological properties and protective efficacy of a single mycobacterial antigen displayed on polyhydroxybutyrate beads
Source: Microb Biotechnol. 2017 Jul 17;10(6):1434–40. doi: 10.1111/1751-7915.12754 (PMC5658617; doi:10.1111/1751-7915.12754)
Supplement: Supplementary file 1 — Fig. S1. IgG1 and IgG2c titres, expressed as EC50 values in mice (8 per group) vaccinated subcutaneously three times at 9 day intervals with different doses of Rv1626 displayed on beads and Wt beads. Fig. S2. Serum IgG1 and IgG2c titres, expressed as EC50 values in mice (8 per group) vaccinated subcutaneously three times at 9 day intervals with Rv1626 beads or Rv1626 beads displaying different immune modulators. Fig. S3. Cytokine responses of mice splenocytes upon stimulation with soluble Rv1626 (recRv1626) and analysed by cytometry bead array. [file MBT2-10-1434-s001.pdf]

Supplementary Information

Immunological properties and protective efficacy of a single mycobacterial antigen displayed on polyhydroxybutyrate beads

Patricia Rubio-Reyes, Natalie A. Parlane, Bryce M. Buddle, D. Neil Wedlock, Bernd H.A. Rehm

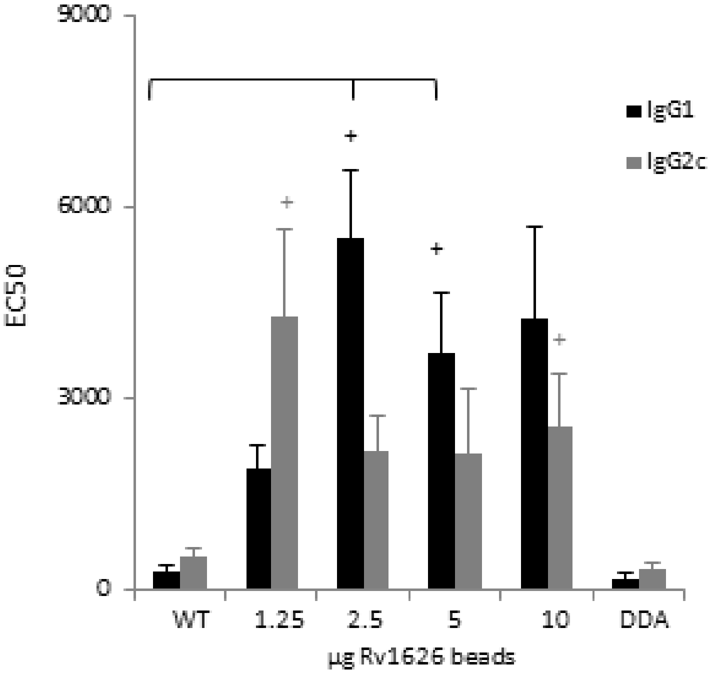

Figure S1: IgG1 and IgG2c titres, expressed as EC50 values in mice (8 per group) vaccinated subcutaneously three times at 9 day intervals with different doses of Rv1626 displayed on beads and Wt beads. At 3 weeks after the final vaccination, antibody responses to soluble recombinant Rv1626 were analysed by ELISA according to methods described by Rubio-Reyes et al., 2016. Each data point represents the mean ± standard error of the mean, +, significantly greater than DDA vaccinated group; groups between brackets, significantly different. ($p<0.01$).

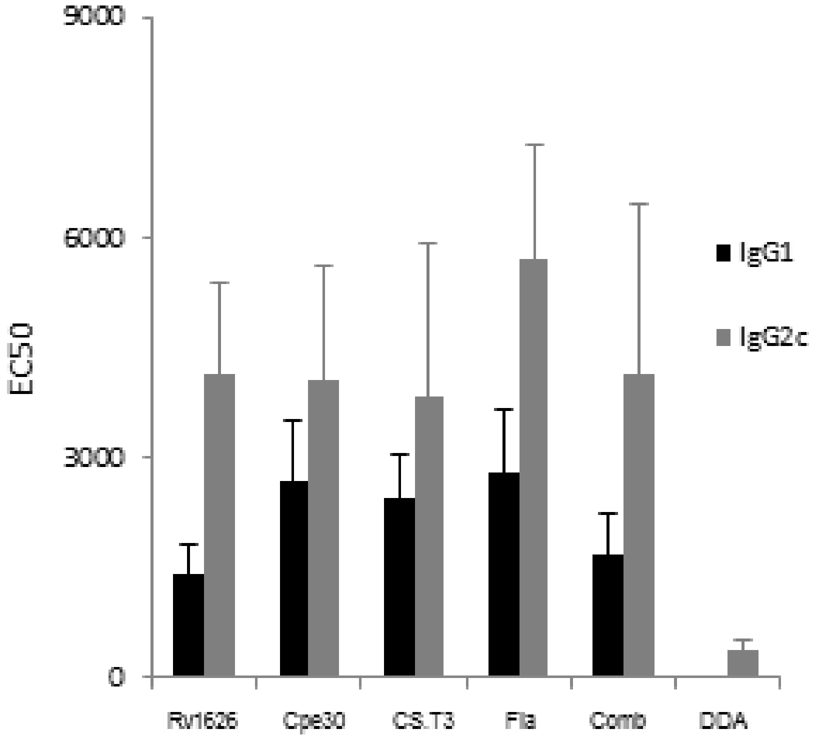

Figure S2: Serum IgG1 and IgG2c titres, expressed as EC50 values in mice (8 per group) vaccinated subcutaneously three times at 9 day intervals with Rv1626 beads or Rv1626 beads displaying different immune modulators. Three weeks after the final vaccination, Antibody responses to soluble recombinant Rv1626 were analysed by ELISA according to methods described by Rubio-Reyes et al., 2016. Each data point represents the mean ± standard error of the mean.

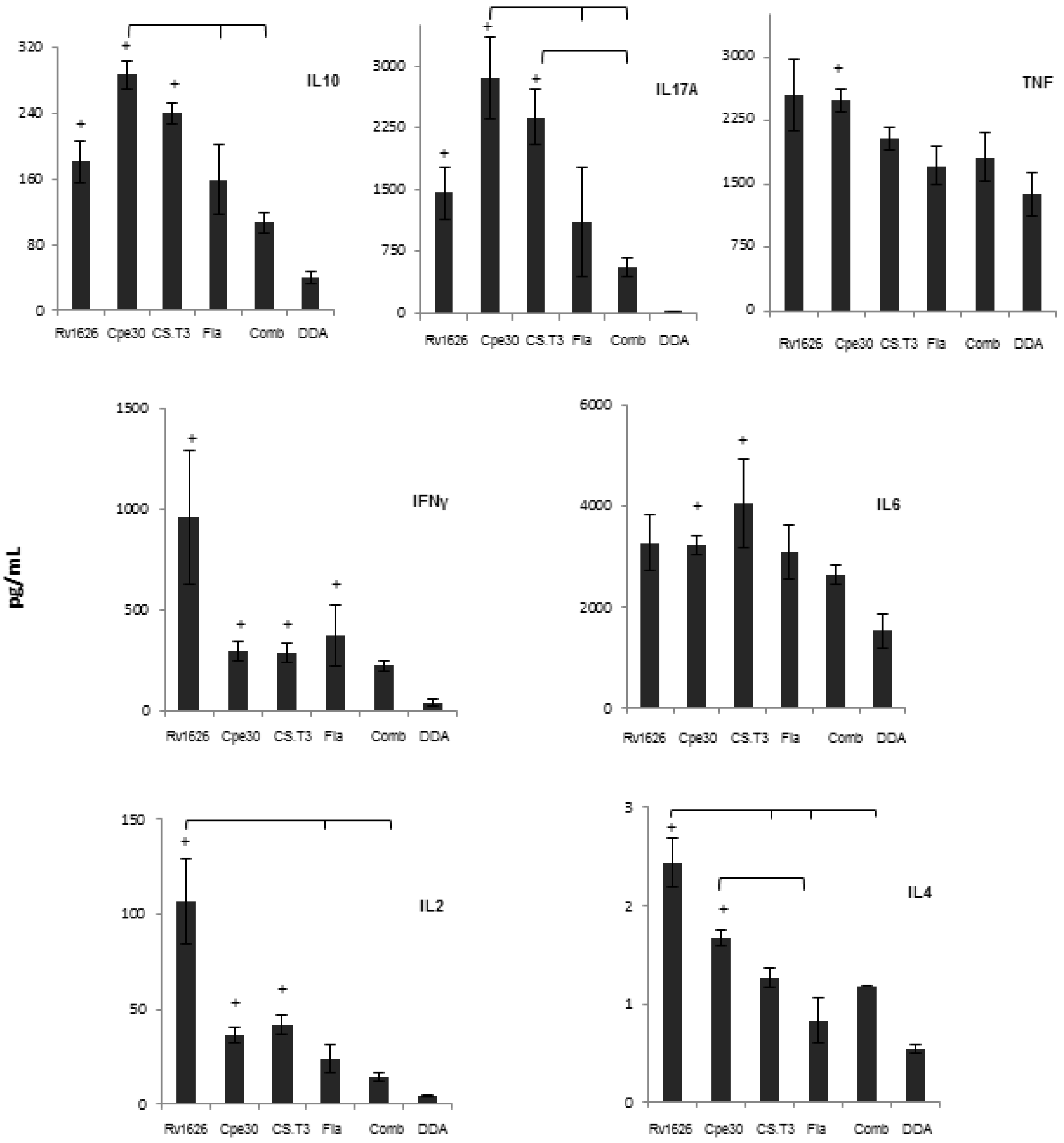

Figure S3: Cytokine responses of mice splenocytes upon stimulation with soluble Rv1626 (recRv1626) and analysed by cytometry bead array. Mice (8 per group) were vaccinated subcutaneously three times at 9 day intervals with Rv1626 beads or immune modulators-Rv1626 beads. Three weeks after the final vaccination, mice were euthanized and splenocytes obtained and cultured as described by Parlane et al., 2012. Cytokine release was measured in the splenocyte culture supernatants. Each data point represents the mean ± standard error of the mean, +, significantly greater than DDA vaccinated group; groups between brackets, significantly different. ($p<0.01$).
